# Supplementary material for: Transcriptional dynamics of gametogenesis in the green seaweed Ulva mutabilis identifies an RWP-RK transcription factor linked to reproduction
Source: BMC Plant Biol. 2022 Jan 6;22:19. doi: 10.1186/s12870-021-03361-3 (PMC8734247; doi:10.1186/s12870-021-03361-3)
Supplement: Supplementary file 2 — Additional file 2. [file 12870_2021_3361_MOESM2_ESM.docx]

Supplementary figures


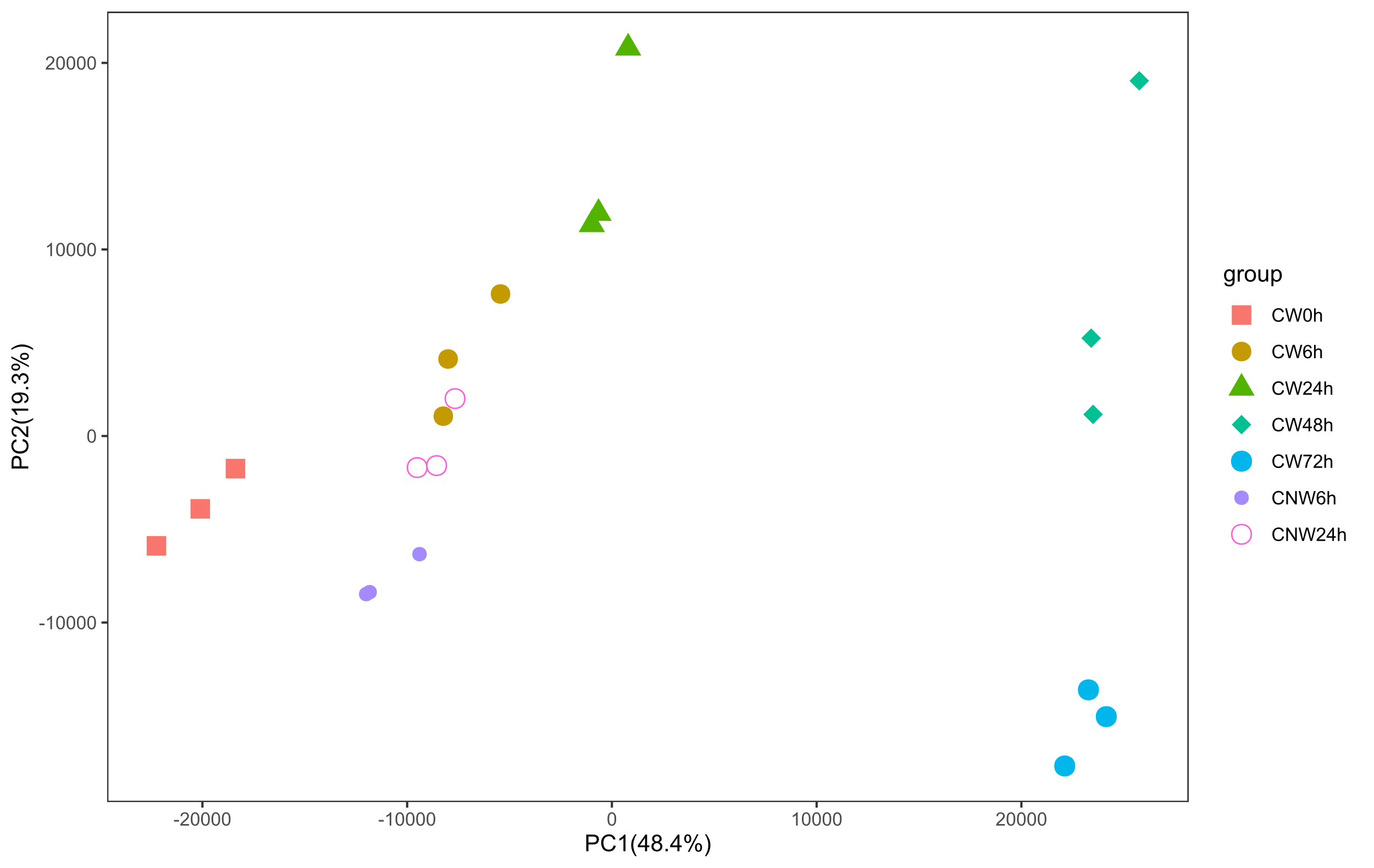


Figure S1. PCA analysis for the all the samples. Three replicates of each group are indicated by same color and shape.


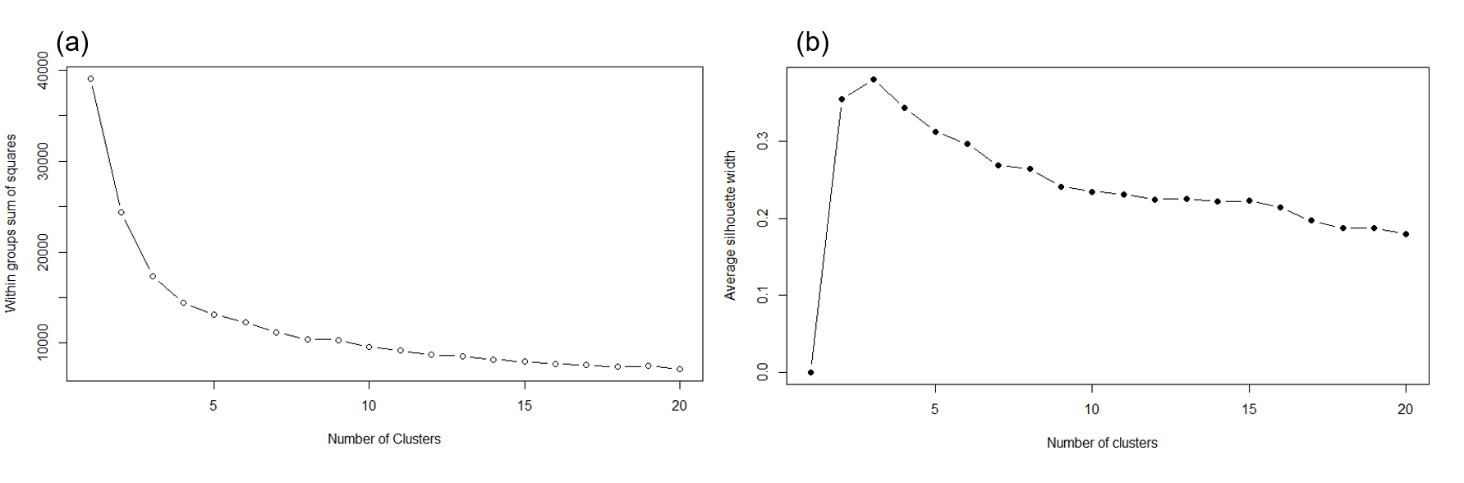


Figure S2. Analysis for the optimal number of the clusters for DEGs. (a): The sum of the squared distance between each member of a cluster and its cluster centroid (SSE) analysis. (b): The silhouette value analysis describes how similar a gene is to its own cluster compared to other clusters.
